# Small-volume plan optimization of inoperable early-stage centrally-located non-small-cell lung cancer using VMAT-based SBRT under the DIBH scenario: a single-arc model or a dual-arc plan?

**DOI:** 10.3389/fonc.2026.1718548

**Published:** 2026-01-23

**Authors:** Yangyang Huang, Jinjin Yuan, Alan Chu, Jun Yang, Yibao Liu

**Affiliations:** 1 Department of Radiotherapy, The Second Affiliated Hospital of Zhengzhou University, Zhengzhou, Henan, China; 2 School of Nuclear Science and Engineering, East China University of Technology, Nanchang, Jiangxi, China; 3 Department of Radiotherapy, The Third Affiliated Hospital of Zhengzhou University, Zhengzhou, Henan, China

**Keywords:** BOT, DIBH, inoperable early-stage centrally-located NSCLC, SA and DA plans, SBRT, small-volume, VMAT

## Abstract

**Introduction:**

This study aimed to comprehensively analyze the dosimetric parameters, plan complexity, gamma passing rates (GPRs), and most importantly, the beam-on time (BOT) of stereotactic body radiotherapy (SBRT) for small-volume inoperable early-stage centrally-located non-small-cell lung cancer (NSCLC) at a radiotherapy center. The analysis was based on both single-arc (SA) and dual-arc (DA) VMAT techniques under the deep inspiration breath hold (DIBH) scenario.

**Methods:**

We retrospectively selected 24 cases of small-volume inoperable early-stage centrally-located NSCLC treated with SBRT under the DIBH scenario at our institution between March 2021 and June 2024. The redesigned SA-VMAT plans (SA plans) adopted the same prescription dose of 50 Gy/5 fractions and flattening-filter free (FFF) beam as the original DA-VMAT plans (DA plans). The 2-group plans (i.e., the SA and DA plans) were normalized to cover 95% of the planning target volume (PTV) and 99% of the gross tumor volume (GTV) by the prescription dose. The evaluation factors included PTV parameters (D_98%_, D_2%_, HI, CI, and R_50%_), organs at risk (OARs), plan complexity (segments and MUs), GPRs, and BOT.

**Results:**

The SA technique consistently yielded superior plans. Among the PTV parameters, the SA plans were superior to the DA plans in D_98%_, D_2%_, and HI (all *p* < 0.05), whereas the CI and R_50%_ of the 2-group plans were comparable (all *p* > 0.05), and the SA plans had an increase in the ipsilateral PBT D_max_ (*p* < 0.05). Otherwise, the differences between other OARs were insignificant (all *p* > 0.05). The SA plans had reduced complexity, with mean segments and mean MUs decreasing by 18.82% and 8.15%, respectively (all *p* < 0.001); the GPRs did not differ significantly under the three acquisition parameters (all *p* > 0.05). The mean BOT was reduced by 19.70% in SA plans (*p* < 0.001).

**Discussion:**

The SA plans significantly shortened the BOT while maintaining comparable plan quality, thereby improving comfort for patients with small-volume inoperable early-stage centrally located NSCLC under the DIBH scenario. Future studies should accumulate more patient data to evaluate the long-term clinical outcomes of SA plans.

## 1 Introduction

In recent years, with the aging of the population and advancements in screening techniques, an increasing number of patients with inoperable early-stage centrally-located non-small-cell lung cancer (NSCLC) have been identified in clinical practice (1, 2). Centrally-located NSCLC is defined as tumors where the closest point is within 2 cm of (but not abutting) the proximal bronchial tree (PBT) or within 2 cm of (whether abutting or not) the mediastinal structures (3). Stereotactic body radiotherapy (SBRT) is increasingly recognized as a key treatment modality for inoperable early-stage centrally-located NSCLC (4). SBRT is characterized by a small number of fractions, high fractional dose, high conformality, and accurate delivery (5). Studies have shown that SBRT can safely treat patients with early-stage centrally-located NSCLC and achieve favorable outcomes (3, 6–8).

A major limitation of early-stage centrally-located NSCLC SBRT treatment is the dose to mediastinal organs at risk (OARs) and healthy lungs, with the radiotoxicity being most prominent in the ipsilateral PBT and healthy lungs (9, 10). This is because the ipsilateral PBT and healthy lungs are near the target volume, and the equivalent biological dose from SBRT is large. Radiotoxicity in the ipsilateral PBT includes fatal hemoptysis (11), and radiotoxicity in the healthy lungs includes fatal radiation pneumonitis in patients with poor respiratory function, particularly those with underlying interstitial lung disease (4). Therefore, it is essential to strictly control the dose of ipsilateral PBT and healthy lungs according to the relevant standard (3), which requires using the most potent modulation technique. Volumetric-modulated arc therapy (VMAT) is the most powerful modulation technique of all linac-based SBRT treatments, providing the best possible OAR sparing (12–14), as well as a significant decrease in treatment time (15, 16). However, the VMAT technique is susceptible to respiratory motion and produces interplay effects (17). Interplay effects can lead to hot and cold spots within the target volume, and dose variations of up to 10% in a single fraction have been observed (18). Compared to conventional treatments, SBRT has fewer fractions and a smaller target volume, making it more susceptible to interplay effects (19). To mitigate these effects, respiratory management strategies are crucial, and the deep inspiration breath hold (DIBH) technique is one of the most widely used approaches (20, 21).

The DIBH technique relies on specific tools such as the Active Breathing Coordinator (ABC) and Optical Surface Monitoring System (OSMS) to achieve respiratory control, thereby reducing interplay effects and the radiation dose to OARs (22). Under the DIBH scenario, patients attempt a maximal inspiratory volume during the simulations and the treatments, which allow the mediastinal OARs, such as the ipsilateral PBT, the healthy lungs, the esophagus, and the heart, to be removed from the high-dose volume (23) and potentially increase the target dose without increasing the OAR radiotoxicity (24). Ottosson et al. (22) observed that in the lung cancer radiotherapy, DIBH scenario compared to the free-breathing state, patients had an 86.8% increase in bilateral lung volume, a 14.8% decrease in gross tumor volume (GTV), an 11.6% and 19.9% decrease in bilateral V_5Gy_ and V_20Gy_, respectively, and a 12.6% decrease in mean cardiac dose. Panakis et al. (25) demonstrated that, compared to the free-breathing state, the use of the ABC reduced D_mean_, V_20Gy_, and V_13Gy_ by 25%, 21%, and 18%, respectively, in healthy lungs.

However, the DIBH scenario requires patients to attempt a maximal inspiratory volume during simulations and treatments and to hold their breath for a sufficient period; therefore, minimizing the beam-on time (BOT) during treatment may improve patients’ comfort and the feasibility of the DIBH technique (26). In addition to using the VMAT technique to minimize BOT, it is necessary to use a high-dose-rate flattening filter-free (FFF) beam (27). Apart from shortening treatment time, the FFF beam offers other advantages, such as a smaller penumbra and reduced head scatter (28–30),, which result in a lower out-of-field dose (31, 32). Traditionally, the dual-arc VMAT plans (DA plans) have been used to treat lung cancer (33–35). However, DA plans still require a relatively long BOT and do not represent the minimum achievable BOT.

Based on years of clinical experience, one approach to shorten the BOT of VMAT-SBRT without compromising the plan quality is to reduce the number of arcs. This is achieved through the rational adjustment of segment sequencing parameters, resulting in a remarkable reduction in BOT. Multiple pieces of literature have reported the successful application of single-arc VMAT (SA) plans in the head and neck, pelvis, and many other tumor sites (36–39). Ning et al. (39) found that when comparing the plans of 20 patients with nasopharyngeal carcinoma of DA plans, SA plans reduced the BOT by 29.8% without sacrificing target coverage, conformity index (CI) and homogeneity index (HI). Panizza et al. (38) analyzed 20 prostate cancer cases and showed that, compared with DA plans, SA plans significantly reduced the average doses to the rectum and bladder; target coverage and HI were comparable, while BOT was decreased by 22%.

However, the application of SA plans in DIBH radiotherapy for small-volume early-stage centrally-located NSCLC has rarely been reported. This study aims to explore the feasibility of SA plans for small-volume inoperable early-stage centrally-located NSCLC under the DIBH scenario by comparing the dosimetric parameters, plan complexity, gamma passing rates (GPRs), and most importantly, the BOT of SBRT treatment between SA and DA plans.

## 2 Methods

### 2.1 Patient cohort

Twenty-four consecutive patients with small-volume inoperable early-stage centrally-located NSCLC treated under ABC-DIBH scenario with SBRT were retrospectively selected from March 2021 and June 2024. This study was approved by the Ethics Committee of the Second Affiliated Hospital of Zhengzhou University (ethics number: 2023202), and written informed consent was obtained from the patients to use their anonymized data. All methods were carried out in accordance with relevant guidelines and regulations. The original DA plans were all designed using a 50Gy/5 fractions prescription and a 6 MV-FFF beam. The general characteristics of the patients are shown in **Table 1**.

**Table 1:** Summary of patients’ general characteristics.

Type	Description
Staging	9 cases T1N0M0, 15 cases T2N0M0
Age	47–71 years
Sex	13 males, 11 females
Tumor location	10 cases in the lower lobe of the left lung, 14 cases in the lower lobe of the right lung
Tumor size	PTV median 23.56 cc, ranging from 8.52 to 40.66 cc

In accordance with the RTOG 0236 and 0813 protocols (3, 40), patients were screened with the following criteria: tumors (planning target volume, PTV) no larger than 5 cm and located outside the 0.5 cm area around the PBT and within the 2 cm area or immediately adjacent to the mediastinal or pericardial pleura. Patients with tumor extent that could not be defined on CT (e.g., tumors surrounded by solid lesions or pulmonary atelectasis) and patients with ultracentral tumors were excluded (41, 42). Moreover, in light of the clinical practice at our center, patients with inoperable early-stage NSCLC that is centrally located and whose tumors have a volume exceeding 50 cc have not been subjected to the five-fraction SBRT regimen. The primary reason for this was the concern over the occurrence of severe radiation toxicities like esophagotracheal fistula.

### 2.2 Positioning and contouring

Each patient underwent a spiral CT scan using a big-bore CT scanner (Philips Medical Systems, Cleveland, OH) with a slice thickness of 3 mm. All patients were placed in the supine position with arms raised and hands grasping the T-handle. The chest was immobilized using a vacuum bag (Guangzhou Klarity Medical Equipment Co., Ltd., Guangzhou, China) in conjunction with a surface-guided radiotherapy (SGRT) system, and radiotherapy was performed under the guidance of the SGRT system to ensure the positional accuracy of patients during treatment. The scanning area encompassed all OARs that were subject to evaluation, along with an extra 15 cm in both the cranial and caudal directions respectively (43).

According to the RTOG recommendations (3, 44), heterogeneity corrections were required for planning, and the GTV was delineated on lung windows. The PTV was created by adding an isotropic 5 mm margin to the GTV. The OARs that needed to be delineated included the healthy lungs (ipsilateral lung and lung all that exclude the GTV), the ipsilateral PBT, the spinal cord, the esophagus, the great vessels, the heart, the ipsilateral brachial plexus, and the skin.

### 2.3 Planning

Based on the Monaco 5.4 (Elekta AB, Stockholm, Sweden) treatment planning system, a new SA plan was designed to replicate the corresponding DA plan to ensure that the cost function and weights of the 2-group plans (i.e., the SA and DA plans) per patient remained constant. All plans were designed with a prescription of 50Gy/5 fractions and a 6MV-FFF beam. All plans were designed so that the dose constraints for the ipsilateral PBT and the healthy lungs needed to be met first, even prior to the target coverage. All plans were rescaled to meet the coverage requirement, specifically ensuring that 95% of the PTV and more than 99% of the GTV covered by the prescription dose. Moreover, all hot spots (with the dose range between 120% and 150% of the prescription dose) were positioned within the GTV. If OARs overlapped with the PTV or GTV, the minimum dose for the PTV or GTV in the overlapping volume should be at least 70% and 90% of the prescription dose, respectively. All plans were delivered using an Infinity accelerator equipped with an Agility collimator featuring 80 pairs of leaves and an isocenter projection thickness of 5 mm.

To maximize compliance with the OAR dose constraints, each of the 2-group plans was designed with one coplanar and one noncoplanar beam: one consisted of a 210-240° arc (table angle 0°) on the ipsilateral side, and the other of a 40-60° anterior arc (table angle 90°). The specific geometric parameters for the beam at a table angle of 0° are as follows: for left lung tumors, the counterclockwise arc has a start angle of 180° and an end angle ranging from 330° to 300°; for right lung tumors, the clockwise arc has a start angle of 180° and an end angle ranging from 30° to 60°. By contrast, for the beam at a table angle of 90°, the clockwise arc is configured with a start angle of 330-340° and an end angle of 20-30°, irrespective of whether the tumor is located in the left or right lung. All beam angles should be ascertained by means of conducting trial runs of the collision test in light of the patients’ body shapes. The arc increment was set at 10°, and the collimator angles ranged from -20° to +20°. These collimator angles were adjusted to be different from each other so as to minimize the tongue-and-groove effect (45). The isocenters for the 2-group plans were placed at the center of the PTV. Dose calculations for the 2-group plans were carried out by employing the Monte Carlo algorithm, with a dose grid resolution of 2.0 mm and a statistical uncertainty of 1% per plan. To minimize variables, the 2-group plans adopted the same PTV and OAR optimization constraints. In the segment optimization parameters, the SA plans were configured with one beam and one arc, having a maximum of 200 segments per arc, a minimum segment width of 0.50 cm, and applying low fluence smoothing. Meanwhile, the original DA plans were set to feature one beam and two arcs, with a maximum of 120 segments per arc, a minimum segment width of 1.0 cm, and adopting medium fluence smoothing.

### 2.4 Plan evaluation factors

Dosimetric parameters for PTV and OARs were evaluated for the newly optimized SA plans and compared with the DA plans.

The PTV evaluation parameters included D_98%_, D_2%_, HI, CI, and R_50%_, where D_98%_ and D_2%_ represent the approximate minimum and approximate maximum dose of the PTV, respectively. According to the RTOG 0813 protocol, D_2%_ decreases with decreasing PTV. At PTV = 50 cc, D_2%_ < 77.0 Gy; at PTV < 10 cc, D_2%_ < 57.0 Gy. D_98%_ > 45.0 Gy. HI describes the dose homogeneity within the PTV (46), with HI = D_5%_/D_95%_, where D_5%_ and D_95%_ represent the dose received by greater than 5% and greater than 95% of the PTV, respectively. Because the pursuit of PTV dose homogeneity will increase the OAR dose, the maximum HI must be set carefully when the PTV does not contain tissues that need to be retained functionally (47). HI < 1.6 is generally considered appropriate based on the radiotherapy center’s situation. CI and R_50%_ were used to compare the high and intermediate dose spillage between the 2-group plans (48). CI = PIV/TV, where PIV is the prescription isodose volume, and TV is the target volume. CI takes a value ≥ 1; the closer the CI is to 1, the better. According to the RTOG 0813 protocol, CI is < 1.5, preferably < 1.2. R_50%_ represents the ratio of the 50% prescription isodose volume to the PTV, with R_50%_ increasing as the PTV decreases. According to the RTOG 0813 protocol, R_50%_ is < 5.0 for PTV = 50 cc and <7.5 for PTV <10 cc.

OARs were compared in compliance with the RTOG 0813 protocol and other literature (41, 49), including V_5Gy_ < 60% and V_20Gy_ < 20% in the ipsilateral lung and V_5Gy_ < 40% and V_20Gy_ < 10% in the lung all; V_18Gy_ < 4 cc and D_max_ < 52.5 Gy in the ipsilateral PBT; spinal cord V_22.5Gy_ < 0.25 cc, V_13.5Gy_< 0.5 cc and D_max_ < 30 Gy; esophagus V_27.5Gy_ < 5 cc and D_max_ < 52.5 Gy; heart V_32Gy_ < 15 cc and D_max_ < 52.5 Gy; great vessels V_47Gy_ < 10 cc and D_max_ < 52.5 Gy; ipsilateral brachial plexus V_30Gy_ < 3 cc and D_max_ < 32 Gy; skin V_30Gy_ < 10 cc and D_max_ < 32 Gy.

Plan complexity assessment was performed based on segments and MUs, as these two parameters positively correlate with the plan complexity (35). Dose delivery accuracy, i.e., GPRs, was also recorded. Based on the AAPM TG 218 report and other literature (50, 51), and in conjunction with the radiotherapy center, the qualified GPRs were set as 2%/2mm > 95%, 2%/1mm > 85%, and 1%/2mm > 90%, with data below 5% of the maximum dose excluded. BOTs for the 2-group plans were recorded while measuring GPRs using SRS MapCHECK (equipped with StereoPHAN phantom) (Sun Nuclear, Melbourne, FL).

### 2.5 Statistical analysis

The paired Student’s *t*-test (SPSS 25.0, IBM SPSS Statistics for Windows, NY, USA) was used to compare the parameters of SA and DA plans, and a *p* value < 0.05 was considered statistically significant.

## 3 Results

### 3.1 PTV parameters

The PTV median [range] was 23.56 [8.52-40.66] cc. The differences in PTV parameters between the SA and DA plans are shown in **Table 2**. The PTV homogeneity of the SA plans was significantly better than that of the DA plans, which can be seen from the statistically significant differences in D_98%_, D_2%_, and HI between the 2-group plans (all *p* < 0.05). Compared with the DA plans, the SA plans exceeded the PTV D_98%_ by 1.03 Gy, and the D_2%_ and HI were lower by 1.96 Gy and 0.04, respectively. The 2-group plans all met the PTV D_2%_ limit (D_2%_ < 77 Gy) and the HI limit (HI < 1.6). One of the SA plans and two of the DA plans did not meet the PTV D_98%_ limit (D_98%_ > 45Gy). There were no significant differences in CI and R_50%_ between the two groups (all *p* > 0.05), and all CIs met the standard. All but two SA plans and one DA plan met the R_50%_ requirement (R_50%_ < 5.0 for PTV < 50cc).

**Table 2:** Summary of PTV evaluation parameters for the 2-group plans.

Parameters (mean [SD])	SA	DA	Mean difference (95% CI)	*p* value
D_98%_(Gy)	46.54 (2.76)	45.51 (3.05)	1.03 (0.37, 1.69)	0.005*
D_2%_(Gy)	60.59 (2.61)	62.55 (4.04)	-1.96 (-3.15, -0.56)	0.008*
HI	1.20 (0.03)	1.24 (0.06)	-0.04 (-0.06, -0.01)	0.004*
CI	1.14 (0.06)	1.13 (0.04)	0.01 (-0.02, 0.02)	0.919
R_50%_	5.15 (0.66)	5.09 (0.60)	0.06 (-0.01, 0.14)	0.103

SD, standard deviation; SA, single arc; DA, dual arc; PTV, planning target volume; D_98%_, dose to 98% of the target volume; D_2%_, dose to 2% of the target volume; CI, conformity index; R_50%_, the ratio of the 50% prescription isodose volume to the PTV; A statistically signiﬁcant difference result is indicated by an asterisk (*).

### 3.2 OAR sparing

The mean (SD) values of all OAR parameters for the SA and DA plans and the corresponding mean absolute dose difference are presented in **Table 3**. The OAR sparing parameters for the 2-group plans all followed the RTOG 0813 protocol, except for one DA plan that had a slight overdose of V_15Gy_ for ipsilateral PBT (V_15Gy_ = 4.2 cc > 4 cc). In the SA plans, a significant increase in the value of the ipsilateral PBT D_max_ was observed (*p* < 0.05). The mean absolute dose difference was 1.19 Gy (2.47%) for ipsilateral PBT D_max_. For other OARs, in particular, the difference between the ipsilateral PBT V_18Gy_ and the healthy lungs (ipsilateral lung and lung all that exclude the GTV) were not statistically significant (all *p* > 0.05). Some OAR data are not presented in **Table 3** because they were all 0 (spinal cord V_22.5Gy_, ipsilateral brachial plexus V_30Gy_, and skin V_30Gy_) or overwhelmingly 0 (spinal cord V_13.5Gy_, esophagus V_27.5Gy_).

**Table 3:** Summary of OAR evaluation parameters for the 2-group plans.

Parameters (mean [SD])		SA	DA	Mean difference (95% CI)	*p* value
Ipsilateral PBT	V_18Gy_ (%)	3.24 (0.92)	3.14 (0.96)	0.10 (-0.04, 0.24)	0.142
	D_max_ (Gy)	48.24 (5.26)	47.05 (4.59)	1.19 (0.02, 2.36)	0.047*
Ipsilateral lung	V_5Gy_ (%)	34.83 (7.22)	35.40 (7.17)	-0.57 (-1.99, 0.85)	0.404
	V_20Gy_ (%)	8.04 (3.16)	7.98 (3.17)	0.06 (-0.05, 0.18)	0.244
Lung all	V_5Gy_ (%)	19.36 (1.32)	19.39 (5.58)	-0.03 (-0.49, 0.44)	0.911
	V_20Gy_ (%)	4.03 (1.66)	4.00 (1.68)	0.03 (-0.02, 0.08)	0.264
Spinal cord	D_max_ (Gy)	8.40 (2.38)	9.12 (2.26)	-0.72 (-1.64, 0.19)	0.112
Esophagus	D_max_ (Gy)	18.10 (10.08)	18.88 (9.74)	-0.78 (-2.19, 0.64)	0.258
Heart	V_32Gy_ (%)	1.46 (2.22)	1.62 (2.43)	-0.16 (-0.33, 0.02)	0.072
	D_max_ (Gy)	36.55 (13.75)	36.67 (13.87)	-0.12 (-1.45, 1.22)	0.858
Great vessels	V_47Gy_ (%)	0.09 (0.22)	0.09 (0.21)	-0.00 (-0.01, 0.02)	0.603
	D_max_ (Gy)	44.53 (7.02)	44.89 (7.02)	-0.36 (-1.06, 0.35)	0.298
Ipsilateral brachial plexus	D_max_ (Gy)	0.35 (0.28)	0.36 (0.27)	-0.01 (-0.03, 0.02)	0.955
Skin	D_max_ (Gy)	13.34 (1.73)	13.66 (1.35)	-0.32 (-1.17, 0.52)	0.429

SD, standard deviation; SA, single arc; DA, dual arc; PBT, proximal bronchial tree; V_x Gy_, the ratio of the volume received > x Gy dose by an organ to the total volume; D_max_, the maximum point dose to an organ. A statistically significant difference result is indicated by an asterisk (*).

**Figure 1:**
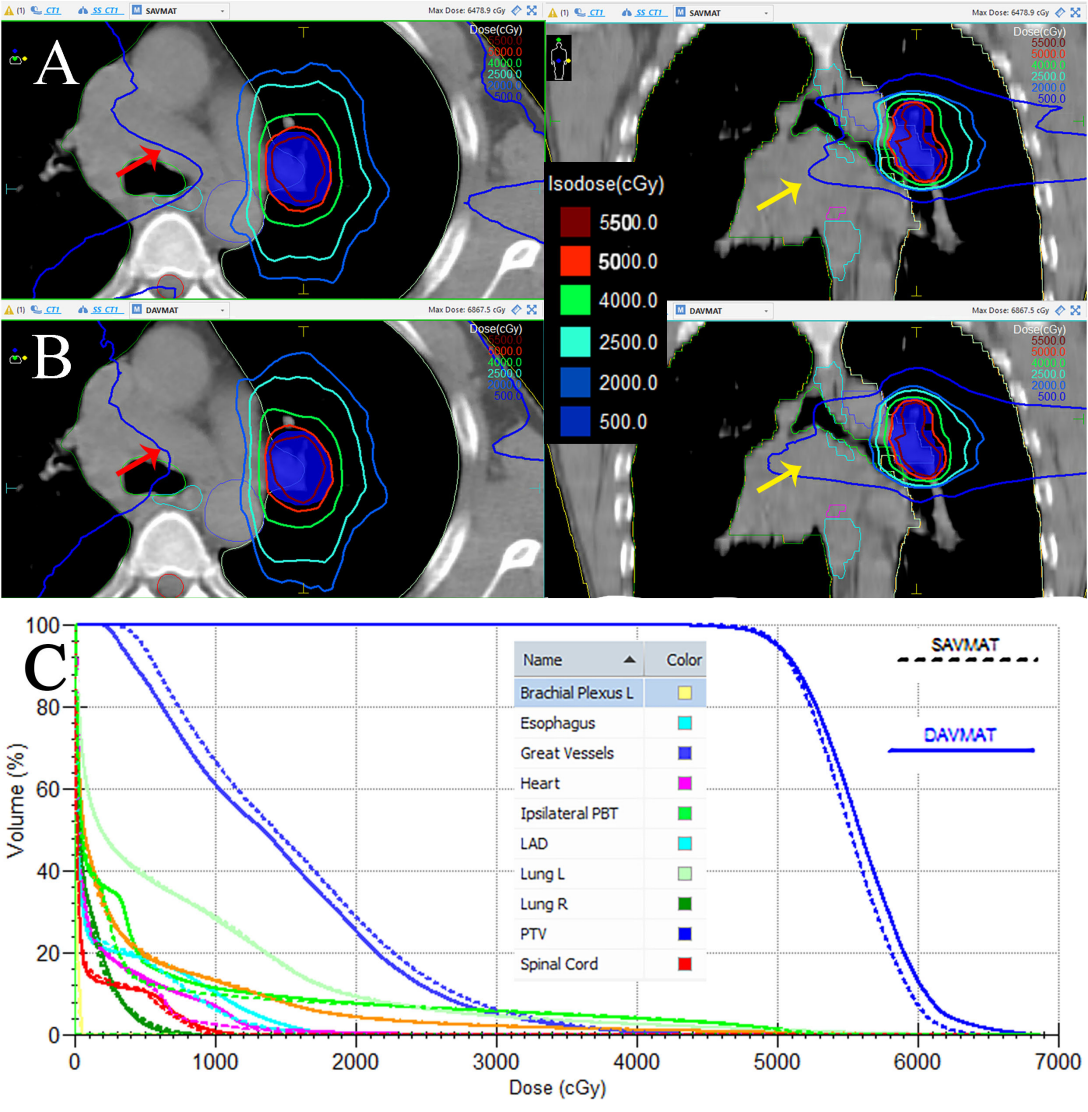
Comparison between SA **(A)** and DA **(B)** dose distribution in the axial (left) and coronal (right) planes, with isodoses ranging from 500cGy to 5500 cGy. **(C)** DVH comparison between the 2-group plans of the Brachial Plexus L, Great Vessels, Heart, Ipsilateral PBT, Lung L, Lung R, PTV, and Spinal Cord for SA (dashed line) and DA (solid line) plans of the same case. The low-dose line (500cGy) of the SA plan showed a certain degree of retraction, while the other dose lines were comparable to those of the DA plan. The red and yellow arrows indicate the changes in the axial and coronal planes, respectively.

Referring to the PTV and OAR sparing parameters, the SA technique produced plans that followed the RTOG 0813 protocol in all cases. **Figure 1** compares the dose distributions, and DVH of SA and DA plans for the same case.

### 3.3 Plan complexity, the GPRs and BOT

Segments and MUs describe the plan complexity. As shown in **Table 4**, the segments of SA plans were significantly reduced compared to DA plans [132.07 (2.10) vs. 156.93 (13.12), *p* < 0.001], with an average reduction of 18.82%; similarly, the MUs of SA plans were significantly reduced compared to DA plans [3090.87 (419.90) vs. 3342.79 (498.02), *p* < 0.001], with an average reduction of 8.15%. Thus, the complexity of SA plans decreased.

**Table 4:** Summary of evaluation parameters for the 2-group plans.

Parameters (mean [SD])		SA	DA	Mean difference (95% CI)	*p* value
Segments	N/A	132.07 (2.10)	156.93 (13.12)	-24.86 (-31.81, -17.92)	<0.001*
MUs	N/A	3090.87 (419.90)	3342.79 (498.02)	-251.92 (-370.99, -132.85)	<0.001*
BOT (s)	N/A	146.78 (22.47)	182.80 (24.97)	-36.02 (-42.65, -29.38)	<0.001*
GPRs (%)	(2%/2mm)	99.29 (0.49)	98.87(1.36)	0.38 (-0.46, 1.29)	0.326
	(2%/1mm)	92.48 (2.87)	90.59 (3.32)	1.89 (-0.54, 4.32)	0.118
	(1%/2mm)	97.39 (1.40)	97.31 (1.76)	0.05 (-1.14, 1.30)	0.890

SD, standard deviation; SA, single arc; DA, dual arc; MUs, monitor units; BOT, beam-on time; N/A, not applicable; GPRs, gamma passing rates. A statistically significant difference result is indicated by an asterisk (*).

Despite the reduced complexity, there were no significant differences in GPRs between the SA plans and DA plans under different acquisition parameters (all *p* > 0.05). All plans had qualified GPRs under different acquisition parameters.

**Figure 2:**
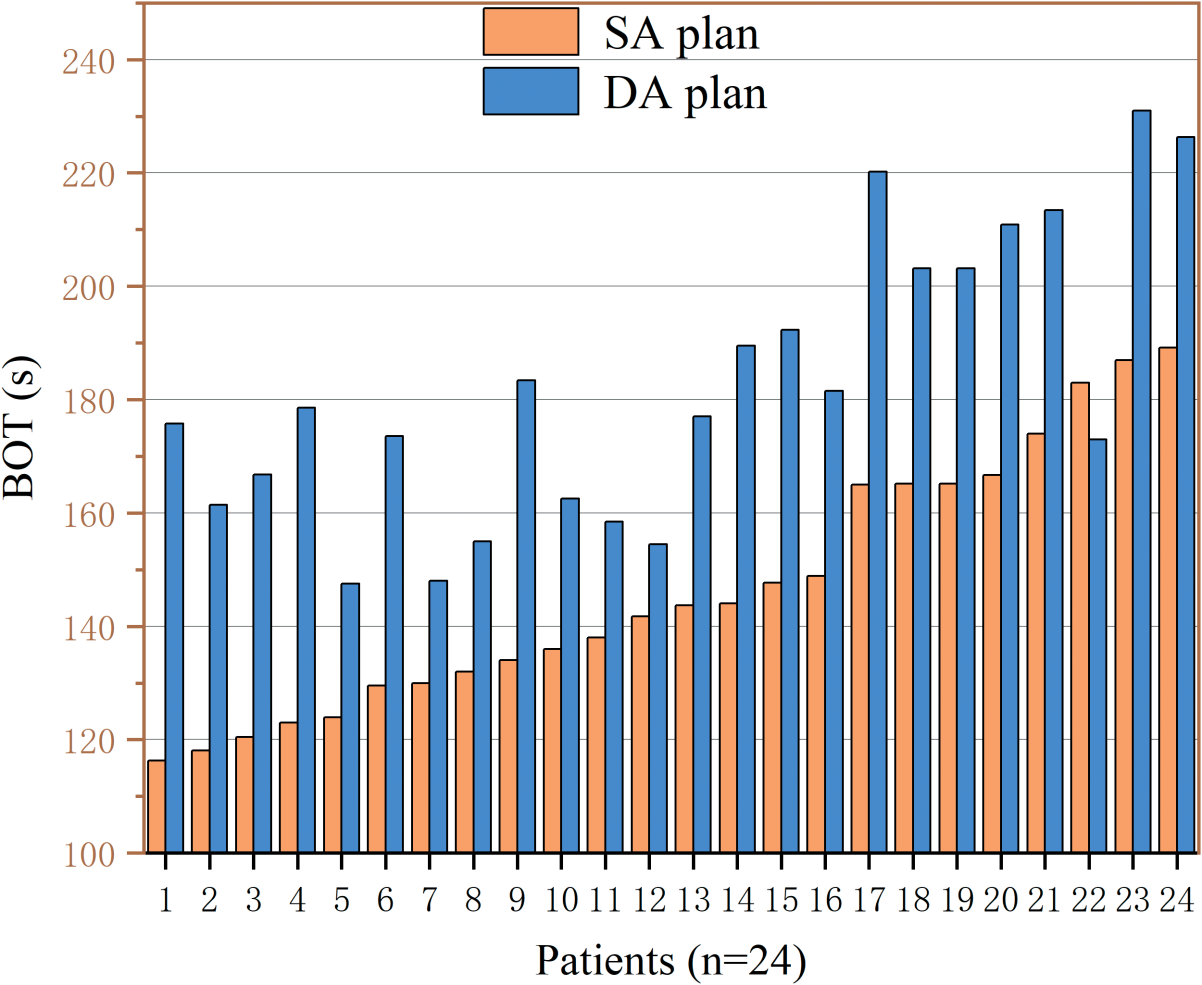
BOTs of each patient for the SA (orange-colored) and DA (blue-colored) plans.

The mean BOT of the SA plans were significantly shorter [146.78 (22.47) vs. 182.80 (24.97), *p* < 0.001], with a mean reduction of 19.70%. The maximum BOT of the SA plans were 189.20 s, whereas the maximum BOT of the DA plans were 226.33 s. **Figure 2** shows the difference in the per-patient BOT of the 2-group plans, and it can be seen that all SA plans had a smaller BOT than the corresponding DA plans.

## 4 Discussion

This study compared dosimetric parameters, plan complexity, the GPRs, and most importantly, the BOT in 24 cases of small-volume inoperable early-stage centrally-located NSCLC treated with SBRT based on SA and DA techniques under the DIBH scenario. Both techniques provided treatment plans that complied with the RTOG 0813 protocol for patients. Dosimetric analysis confirmed the feasibility of the SA plans, characterized by more homogeneous PTV doses and comparable OAR sparing to the DA plans. This is in keeping with our aim of providing superior OAR sparing, such as for the ipsilateral PBT and healthy lungs. Compared to the DA plans, the decreased complexity of the SA plans did not significantly improve GPRs. However, the SA plans provided significant BOT reduction, and BOT reduction is one of the most desirable advantages under the DIBH scenario.

In general, SA plans have shorter treatment times than DA plans (38, 39, 52). However, different literatures have different opinions on the dosimetric quality of SA plans. Zhao et al. (52) suggested that in postoperative VMAT radiotherapy for breast cancer, SA plans had some disadvantages in target dosimetric parameters (CI and HI) compared with DA plans (*p* < 0.05). However, there was no difference between the two in terms of OAR sparing (*p* > 0.05). Ning et al. (39) proposed that in VMAT radiotherapy for nasopharyngeal cancer, the SA plans showed no significant change in PTV coverage and normal tissue dose compared to the DA plans. Panizza et al. (38) compared the advantages and disadvantages of the SA and DA plans for prostate cancer radiotherapy and found that the SA plans were more advantageous overall. The SA plan had significantly lower mean rectal and bladder doses and achieved comparable target coverage and homogeneity.

Guckenberger et al. (53) designed SA and DA plans for prostate, pharyngeal, and paranasal sinus cancer, respectively, and discovered that the SA plan quality was related to the target complexity. SA plans for simple targets can guarantee the plan quality and shorten the treatment time, while DA plans must be used for complex targets to guarantee the plan quality at the cost of increasing the treatment time. Characteristics of early-stage centrally-located NSCLC targets include small-volume tumor, single tumor, absence of positive lymph nodes and distant metastases, and a more regular shape (3, 44), and thus can be considered as simple targets. This was the initial reason for exploring the application of SA plans in this study. In our study, the PTV D_98%_ exceeded 1.03 Gy, D_2%_ and HI were reduced by 1.96 Gy and 0.04, respectively, in the SA plans, and the OAR parameters were barely statistically different from those of the DA plans (except for a significant increase of 1.19 Gy in the ipsilateral PBT D_max_, *p* < 0.05), which supported and validated our view.

In the radiotherapy of NSCLC, damage to the ipsilateral PBT is an important factor limiting the practice of SBRT (3), and central airway injury is 11 times more likely to occur in central lung cancer than in peripheral lung cancer (54). Another common adverse effect is radiation-induced lung injury. The incidence of grade 3–4 lung injury with self-limiting pneumonitis, dyspnea, fever, and chest pain after SBRT treatment for NSCLC had been reported to be 2.7% -27.0%, and grade 5 and above lung injury was uncommon (55). According to the RTOG 0813 protocol (3), none of the early-stage centrally-located NSCLC patients treated with SBRT in the 10 Gy/fx group had grade 3 adverse events with minimal impact on lung function within the first year, and there was no grade 5 or higher events after one year. This study observed a significant increase in the ipsilateral PBT D_max_ in the SA plans (*p* < 0.05). However, the absolute value exceeded by only 2.47%. None of the differences between the other OAR parameters were statistically significant (all *p* > 0.05). Except for one case of a slight overdose of the ipsilateral PBT V_18Gy_ in the SA plan (V_18Gy_ = 4.2 cc > 4cc), the other OARs were well below the requirements of the RTOG 0813 protocol and, therefore, were not expected to result in severe radiotoxicity.

Regarding plan complexity, the segments and MUs of SA plans were all significantly reduced (all *p* < 0.001) by 24.86 and 251.92, respectively, which may be related to the reduction in the number of arcs. Previous researchers (56, 57) observed that segments and MUs increased significantly with the number of arcs used in VMAT plans. However, more segments and MUs contribute to higher plan quality (52) and do not necessarily lead to lower GPRs (58). In our study, the differences in GPRs between SA plans and DA plans were not significant (all *p* > 0.05), which is consistent with the results obtained in previous studies at other sites (38, 59). Compared with the DA plans, the segments and MUs of the SA plans were reduced by 18.82% and 8.15%, respectively, but the BOT was reduced by 19.70%. This may be because the reduction in BOT is mainly caused by the decrease in the number of arcs (from two to one).

Compared with DA plans, the segment optimization parameters for SA plans were moderately relaxed. The core rationale for this adjustment is that if identical segment optimization parameters were adopted for the 2-group plans, the dosimetric parameters of SA plans would be significantly inferior to those of DA plans, thereby rendering SA plans clinically unfeasible. To maintain plan quality while achieving a moderate reduction in BOT, we appropriately relaxed the constraints on segment optimization parameters for SA plans–a strategy that is highly consistent with the findings reported in existing literature. Previous studies have confirmed that minimum segment width and fluence smoothing degree directly affect plan MUs, which in turn influences the overall plan quality (60, 61). Additionally, other study (62) has indicated that as minimum segment width increases, the dosimetric parameters of PTV gradually deviate from the prescribed dose, with a significant increase in target dose inhomogeneity (*p* < 0.05).

Under the DIBH scenario, a shorter BOT is more conducive to improving patient comfort and the feasibility of implementing the DIBH technique (63–65). Insufficient patient compliance constitutes a major limitation of the DIBH technique. As noted by Mah et al. (66), only half of the lung cancer patients included in their study could successfully perform this method; therefore, minimizing the BOT is a prerequisite for the application of DIBH. Compared with 3D conformal radiotherapy and fixed-field intensity-modulated radiotherapy techniques, VMAT, as a rotational therapy technique, has significantly higher efficiency in lung cancer treatment (13, 67). Specifically, in this study, the BOT of SA plans were, on average, 19.70% (36.02 s) shorter than that of DA plans, with a mean BOT of 146.78 s and a maximum BOT of 189.2 s. Although conventional views (52, 53, 59, 68) have suggested that SA plans have a slightly poorer dose distribution while shortening the BOT, our study demonstrated that SA plans can achieve dosimetric parameters comparable to or even better than DA plans. Therefore, we will adopt SA plans for more small-volume inoperable early-stage centrally-located NSCLC patients treated with SBRT under the DIBH scenario in the future.

The strategy of implementing SA plans under the DIBH scenario has shown promising progress, but there is still much work to be done in future research. Firstly, constrained by the practical experience of our center, the PTV in this study was less than 41 cc, which imposes limitations on the generalizability of SBRT for early-stage centrally-located NSCLC. Secondly, it is essential to amass a greater number of patient cases with the aim of minimizing statistical errors. Lastly, the applicability of SA plans to other cancer types, such as breast cancer and peripheral lung cancer, warrants further investigation, and related studies will be pursued.

## 5 Conclusions

Compared with the DA plans, the SA plans can maintain high-quality PTV parameters while achieving comparable OAR sparing, notably for the ipsilateral PBT and healthy lungs. Furthermore, the SA plans significantly reduce the BOT. These attributes, with the reduction in BOT being particularly prominent, represent the most compelling advantages of SA plans for SBRT in treating small-volume, inoperable early-stage centrally-located NSCLC under the DIBH scenario.

## Data Availability

The raw data supporting the conclusions of this article will be made available by the authors, without undue reservation.
